# Platelet Glycoprotein-Ib (GPIb) May Serve as a Bridge between Type 2 Diabetes Mellitus (T2DM) and Atherosclerosis, Making It a Potential Target for Antiplatelet Agents in T2DM Patients

**DOI:** 10.3390/life13071473

**Published:** 2023-06-29

**Authors:** Muttia Amalia, Meidi Utami Puteri, Fadlina Chany Saputri, Rani Sauriasari, Bambang Widyantoro

**Affiliations:** 1 Doctoral Program, Faculty of Pharmacy, Universitas Indonesia, Kampus UI Depok, Depok 16424, Indonesia; muttia.amalia91@ui.ac.id; 2 Laboratory of Pharmacology-Toxicology, Faculty of Pharmacy, Universitas Indonesia, Kampus UI Depok, Depok 16424, Indonesia; meidiutami@farmasi.ui.ac.id; 3 Faculty of Pharmacy, Universitas Indonesia, Kampus UI Depok, Depok 16424, Indonesia; rani@farmasi.ui.ac.id; 4 National Cardiovascular Center Harapan Kita, Department of Cardiology and Vascular Medicine, Faculty of Medicine, Universitas Indonesia, Jakarta 11420, Indonesia; bambang.widyantoro@pjnhk.go.id

**Keywords:** atherosclerosis, antiplatelet, glycoprotein-Ib (GP1b), platelet, type 2 diabetes mellitus (T2DM)

## Abstract

Type 2 diabetes mellitus (T2DM) is a persistent metabolic condition that contributes to the development of cardiovascular diseases. Numerous studies have provided evidence that individuals with T2DM are at a greater risk of developing cardiovascular diseases, typically two to four times more likely than those without T2DM, mainly due to an increased risk of atherosclerosis. The rupture of an atherosclerotic plaque leading to pathological thrombosis is commonly recognized as a significant factor in advancing cardiovascular diseases caused by TD2M, with platelets inducing the impact of plaque rupture in established atherosclerosis and predisposing to the primary expansion of atherosclerosis. Studies suggest that individuals with T2DM have platelets that display higher baseline activation and reactivity than those without the condition. The expression enhancement of several platelet receptors is known to regulate platelet activation signaling, including platelet glycoprotein-Ib (GPIb). Furthermore, the high expression of platelet GP1b has been reported to increase the risk of platelet adhesion, platelet-leucocyte interaction, and thrombo-inflammatory pathology. However, the study exploring the role of GP1b in promoting platelet activation-induced cardiovascular diseases in T2DM patients is still limited. Therefore, we summarize the important findings regarding pathophysiological continuity between T2DM, platelet GPIb, and atherosclerosis and highlight the potential therapy targeting GPIb as a novel antiplatelet agent for preventing further cardiovascular incidents in TD2M patients.

## 1. Introduction

For the past decades, type 2 diabetes mellitus (T2DM) cases have sharply increased in many countries of all income levels [1,2]. It is reported that T2DM is responsible for over 1 million deaths annually, ranking it as the ninth leading cause of mortality worldwide [1,2]. Projections indicate that the global prevalence of T2DM will continue to rise across all regions of the world, reaching 7079 individuals per 100,000 people by 2030 [1,2]. T2DM is a long-lasting medical condition characterized by insufficient insulin production and function, leading to chronically elevated glucose levels, one of its primary manifestations [3,4,5]. Individuals diagnosed with T2DM are at a high risk of developing cardiovascular diseases due to atherosclerosis, including stroke, acute coronary artery disease (CAD), and peripheral arterial disease (PAD) [3,4,5,6]. It is worth noting that T2DM not only promotes atherosclerosis but also influences its specific pattern [6,7,8]. For instance, metabolic dysregulation in T2DM increases the likelihood of arteries below the knee being affected by lower extremity PAD [6,7,8]. Additionally, T2DM contributes to excessive vascular stiffness, impacting disease progression [6,7,8]. Moreover, T2DM raises the risk of restenosis after interventions such as angioplasty with stent implantation, which are crucial for managing atherosclerosis through vascular therapy [6,7,8].

Atherosclerosis is a long-term inflammatory condition characterized by a disturbed interplay between inflammation and the hemostatic system, primarily involving the platelets, and can lead to serious cardiovascular events [9,10]. In the advanced stages of atherosclerosis, the plaque inside the arteries can become more vulnerable due to the generation of new blood vessels, causing the center of the plaque to become hypoxic [9,10,11]. Moreover, the involvement of inflammation in plaque vulnerability is significant [9,10,11]. Platelets are involved in multiple ways in the progression of atherosclerosis, including the production of the protein junctional adhesion molecule-A (JAM-A), activation of macrophages, and alteration of other immune cells’ functions [10,11]. Additionally, platelets can contribute to foam cell generation, which is the generation of immune cells that take up lipids and release inflammatory mediators and cytokines that promote atherosclerosis in a positive feedback loop [10,11].

Dysregulation of glucose metabolism in T2DM patients promotes an inflammatory environment, resulting in atherosclerosis development [3,4,5,9,10]. In individuals with T2DM, the metabolic condition increases oxidative stress, causing endothelial dysfunction and exacerbating the processes of inflammation, platelet activation, and thrombosis, resulting in atherosclerosis aggravation [3,4,5,9,10,12]. Moreover, inflammation in T2DM will lead to a prothrombotic state, stimulating the actions of platelets, endothelial–leukocyte adhesion molecules, and thromboxane to increase thrombosis [12,13]. Inflammation also generates clot persistence and formation, induces pro-coagulant agent production, and activates platelets [10,11,12,13]. The significance of platelets in vascular obstruction or occlusion is strongly linked with their adherence capacity to injured endothelial cells [10,13,14]. After an injury to the blood vessels, platelets rapidly adhere to the subendothelial cells through adhesion receptors to initiate the healing process [10,13,14]. Activated platelets then recruit more platelets and the expanding plug, resulting in increased thrombin generation [10,13,14]. Cumulatively, platelets play a crucial role in the development of atherosclerosis plaque and the formation of blood clots within the arteries, which can lead to cardiovascular events [9,10]. Considering that diabetic patients are at a higher risk of developing cardiovascular events, it is essential to block one or multiple pathways that regulate platelet activation and aggregation processes [4,5]. This blockade is crucial for reducing the risk of cardiovascular events, including ischemic events such as myocardial infarction, in diabetic patients [15,16,17]. Currently, there are multiple antiplatelet agents that are utilized to prevent and mitigate the risk of ischemic events in diabetic patients [15,16,17]. These include cyclooxygenase-1 (COX-1) inhibitors, ADP P2Y12 receptor antagonists, and platelet glycoprotein (GP) IIb/IIIa inhibitors [15,16,17]. These pharmacological treatments are primarily employed in the prevention and treatment of atherothrombotic disorders [15,16,17].

While these agents have been successful in reducing cardiovascular events in diabetic patients, there are reported limitations associated with the current treatment strategies [16,17]. These include issues such as resistance to aspirin and clopidogrel, as well as severe side effects of GP IIb/IIIa inhibitors [16,17,18,19,20]. As a result, ongoing efforts are being made to address these issues. These efforts involve exploring dose modifications, utilizing adjunctive therapies, and seeking out newer agents to improve treatment outcomes [16,17,18]. Establishing novel antiplatelet agents that can reliably and safely inhibit platelet activation and aggregation processes is considered the most encouraging approach for the future, where personalized antiplatelet drug regimens will be tailored to individual requirements [16,17,18]. This could involve utilizing drugs that specifically target dysfunctional pathways in particular patient groups, such as individuals with diabetes [16,17,18].

The platelet receptor glycoprotein (GP) Ib is a member of the family of leucine-rich repeat (LRR) protein kinases and has been recognized as transmembrane receptor type 1 [21,22]. It is also known as the Cluster of Differentiation 42 (CD42) protein [21,22]. GPIb, a major subunit of the GPIb-IX complex, is the second most prevalent adhesion receptor on platelets [22,23,24]. The GPIb-IX complex is also reported to be important for the hemostatic and prothrombotic functions of platelets [22,23]. It carries out its primary role by starting platelet adhesion when there is high force exerted by blood flow on the walls of blood vessels by binding to von Willebrand factor (vWF) in the subendothelial matrix [10,22,25]. Studies show that inhibiting the GPIbα–vWF binding site gives protective benefits, such as decreased microvascular occlusion, leading to better vascular conditions where blood vessels remain unobstructed [10,25,26]. The enhancement of platelet glycoprotein-Ib (GPIb) expression has been associated with the T2DM condition and has been reported to increase the risk of platelet aggregation, platelet-leucocyte interaction, and thrombo-inflammatory pathology [10,25,27,28,29]. Despite numerous studies demonstrating the link between T2DM and platelet hyperreactivity, research on the role of the platelet receptor GPIb in platelet activation-induced cardiovascular disease in T2DM patients remains limited. Therefore, this study comprehensively discusses GPIb’s role in inducing cardiovascular disease by promoting platelet activation, adhesion, and aggregation during T2DM disease progression. Understanding these pathophysiological mechanisms could lead to novel strategies targeting platelet activation to protect patients with T2DM from developing cardiovascular incidents.

## 2. The Physiology of Platelets

Platelets are non-nucleated blood components identified over 130 years ago [14,30]. It is known that platelets are the main cell that controls thrombosis and mediates myocardial infarction, stroke, and venous thromboembolism (VTE) [14,30]. Those make platelets important for blood vessel homeostasis. The human blood platelet diameter is about 2–4 μm, and its circulation number in healthy individuals approximately ranges from 150 to 350 × 10^9^/L [14,30]. Platelets have a short lifespan, circulating in the blood only for one or two weeks following their elimination in the liver and spleen [14,30]. Platelet production primarily occurs in the bone marrow and is triggered by several transcription factors, including thrombopoietin hormone, to stimulate megakaryocyte development [30,31]. The process is initiated by forming polyploid megakaryocytes [30]. During maturation, megakaryocytes experience endomitotic cell cycles and become larger, resulting from increasing diameter [30,31]. After their differentiation in the bone marrow, megakaryocytes move toward the vascular cavity to be closer to the circulation [30,31]. Platelets have no genomic DNA; they only contain mRNA transcripts, enabling them to generate proteins such as cytokines and interleukins [30,31]. Furthermore, platelets can form microparticles with many bioactive compounds for the coagulation process [30,31].

The platelet’s cytoplasm contains three types of granules [32,33]. The first one is alpha granules, with an approximate number of 50 to 60 per platelet [32,33]. Alpha granules contain blood clotting factors such as vWF, growth factors, fibrinogen, coagulation factors V, XI, XIII, and chemokines [32,33]. The second one is platelet lysosomes, which comprise acid hydrolases and cathepsin D and E and play a role in the degradation of glycosaminoglycans, glycoproteins, and glycolipids [32,33]. These protective functions of lysosomes are fundamental for the extracellular matrix remodeling and regulation of the thrombus [32,33]. The third one is dense granules with an approximate number of 4 to 8 per platelet and consists of adhesion proteins, such as GPIIb/IIIa, GPIb, and P-selectin, as well as high concentrations of serotonin, adenine nucleotides, calcium, and phosphates, which are important in platelet aggregation and vascular contraction [32,33].

Hemostasis, thrombosis, and wound healing are regarded as the platelet’s fundamental functions and are achieved through a complicated activation process, resulting in plug development at the injury site, including vascular injury [34,35]. To avoid hemorrhage when blood vessels are damaged, the adhesive capabilities of platelets must be strictly controlled so the cells can rapidly activate accordingly [34,36]. Simultaneously, preventing unwanted platelet adhesion that can lead to thrombosis is crucial. Platelets have several adhesion molecules with specific individual functions that allow them to work distinctly in hemostatic and inflammatory conditions [34,35]. Furthermore, the platelet membrane encompasses diverse receptors, including glycoproteins, integrins, phospholipids, prostaglandin receptors, adenosine diphosphate receptors, immunoglobulin superfamily adhesion receptors, tyrosine kinase adhesive receptors, G-protein-coupled receptors, and leucine-rich adhesion receptors [34,35].

**Figure 1:**
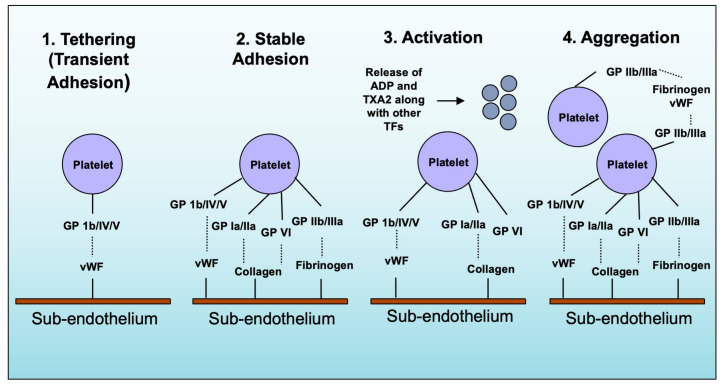
Platelet adhesion, activation, aggregation. When blood vessels are damaged, platelets become activated and attach to the site of damage. This is facilitated by GPIbα and vWF, acting as “tether” and allowing other molecules such as GPVI to interact with collagen. This triggers a series of events that convert integrins on the surface of platelets to a high-affinity state and release ADP and TXA2, further activating platelets and promoting forming a stable blood clot. The tissue factor released from the damaged tissue activates thrombin, causing integrin GPIIb/IIIa to bind with fibrinogen and vWF, further strengthening the attachment of platelets and contributing to the formation of a stable blood clot.

The hemostatic cascade signaling pathway starts with platelet receptors and some ligand interactions expressed on the surface of endothelial cells, in the subendothelial matrix, and as soluble proteins in the bloodstream [9,25,36]. This interaction triggers platelet adhesion and activation, developing thrombi [9,25,36]. GPIb/IV/V, GPVI, and GPIa/IIa receptors are particularly physiologically relevant to platelet adhesion [25,36]. The first step is tathering, the platelet interaction with the exposed endothelial cell matrix (ECM) [25,36]. Especially when blood flows through small arteries and arterioles at high rates, platelets first attach to the ECM by binding platelet GP1b and vWF [25,36]. However, the binding between vWF and GP1b has a quick off-rate and is consequently not enough to mediate firm adhesion [25,36]. Instead, it maintains a close connection between the platelet and the surface, facilitating the interaction between GPVI and collagen [25,36]. The next step is the rolling step. Due to the relatively low-affinity interactions between collagen and GPVI, the platelet GPIa/IIa receptor enhances the platelet interaction with collagen [25,36]. Following stable platelet adhesion, the next step is platelet activation, a paracrine- and autocrine-mediated signaling pathway [25,36]. The process begins with the discharge of thromboxane A2 (TXA2) and adenosine diphosphate (ADP) from platelets, along with the activation of thrombin by tissue factor present in the artery wall [25,36]. This integrates the integrins GPIIb/IIIa with fibrinogen and vWF, reinforcing the firm attachment of platelets and resulting in stable thrombus formation [25,36]. The action of thrombin causes fibrinogen to be changed into fibrin, promoting the growth of the thrombus [9,25]. The summary of platelet adhesion, activation, and aggregation is illustrated in Figure 1.

## 3. Type 2 Diabetes Mellitus (T2DM) Promotes Atherosclerosis by Inducing Platelet Activation

Individuals with TD2M are at great risk of developing cardiovascular disease, which can lead to severe illness and death due to pathological thrombosis resulting from the rupture of atherosclerotic plaque [3,10,27]. Platelets play an important role in initiating and spreading thrombosis [9,10,11]. Recent findings suggest that patients with T2DM exhibit heightened platelet reactivity and basal activation compared to healthy individuals [27,28,29]. Studies have reported that individuals with diabetes have hyperactive platelets that metabolize more quickly, resulting in a faster turnover of platelets [37,38]. This further produces new hyperactive platelets prone to exaggerated responses to stimuli [37,38]. Additionally, platelet counts are higher in T2DM, particularly with an increased number of large platelets representing high reactivity [37,38,39]. The increased platelet hyperreactivity and baseline activation observed in T2DM are known multifactorial phenomena correlated with various biochemical factors, such as high lipid levels (hyperlipidemia), high blood sugar levels (hyperglycemia), insulin resistance, inflammation, and oxidative stress, thereby increasing the cardiovascular risk in T2DM [5,40,41].

Studies have shown that metabolic changes in T2DM can increase platelet activation, which is mostly associated with endothelial cell dysfunction [3,37,41,42,43]. Endothelial cells regulate various processes, including platelet activation and aggregation, vasodilation and vasoconstriction, thrombosis, and fibrinolysis [37,41,42,43]. Endothelial dysfunction, caused by increased blood glucose levels in T2DM, disrupts vascular homeostasis and initiates the atherosclerosis process [37,41,42,43]. Additionally, endothelial dysfunction results in the higher expression of adhesion molecules that promote platelet activation and aggregation, which is crucial in every stage of the atherosclerotic steps [37,41,42,43]. Under normal physiological conditions, endothelial cells produce and release vasodilator substances, such as prostacyclin (PGI2) and nitric oxide (NO), and vasoconstrictor substances, such as endothelin, to maintain vascular tone [37,42,44]. Insulin also affects platelet function by inhibiting P2Y12 signaling and increasing platelet responsiveness to the anti-aggregation effects of NO and PGI2 [17,27,37]. Therefore, a low insulin or insulin resistance level can increase platelet reactivity [5,17].

T2DM is associated with accelerated atherosclerosis due to reactive oxygen species (ROS) production, causing mitochondrial impairment, increased activation of protein kinase C (PKC), and advanced glycation end-products (AGEs) [45,46]. ROS can also stimulate nuclear poly (ADP-ribose) polymerase and switch early glycolytic intermediates into pathogenic pathways [45,46]. These processes lead to reduced mitochondrial biosynthesis, increased ROS production, and interference with the biorhythm of glucose and lipid metabolism [47,48]. The elevated glucose levels in T2DM increase ROS production, decrease NO and PGI2 synthesis through various mechanisms, such as activation of signaling pathways of NF-κB and protein kinase C (PKC), and decrease endothelial NO synthase (eNOS) activity [27,37,44]. These mechanisms result in altered adhesion molecules expression, impaired vasodilation, and advanced vascular inflammation [37,44]. Furthermore, in T2DM, inflammation cytokines are dysregulated and serve as a reciprocal correlation between inflammation and prothrombotic states [49].

Hyperglycemic conditions have also been associated with decreased antioxidant production, such as glutathione, associated with increased TXA2 production, leading to increased platelet activation [37,38]. Moreover, persistent hyperglycemia can increase the glycation of proteins on the platelet surface, causing changes in the activity and signaling of receptor proteins and reducing platelet membrane fluidity [37,50]. 

**Figure 2:**
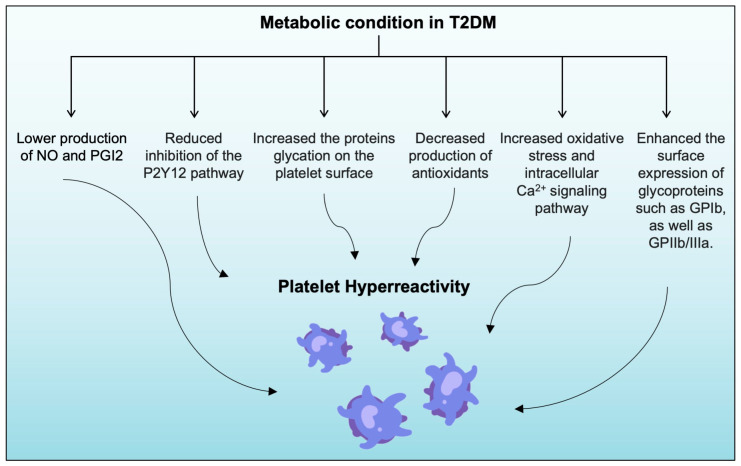
Metabolic dysregulation in T2DM will contribute to greater platelet hyperreactivity. Dysregulated metabolic conditions in patients with T2DM will result in: (1) lower production of NO and PGI2; (2) reduced inhibition of the P2Y12 pathway; (3) increased oxidative stress and intracellular Ca^2+^ signaling activate the PKC pathway; (4) decreased production of antioxidants; (5) increased protein glycation on the platelet surface; (6) enhanced surface expression of glycoproteins, such as GPIb and GPIIb/IIIa. Together, these factors contribute to a prothrombotic environment, thus promoting the development of vascular occlusion and atherothrombosis in T2DM.

Thus, platelets become more responsive to thrombin and aggregate more, increasing platelet adhesion and sensitivity [37,50]. Some evidence also indicates that metabolic changes in patients with T2DM increase the surface expression of specific glycoproteins: GPIIb/IIIa and GPIb [27,28,37]. This increase in expression can lead to greater activation of GPIIb/IIIa, thereby increasing the binding of platelets to vWF and fibrinogen, ultimately leading to greater platelet aggregation. [27,28,37]. The summary of metabolic conditions in T2DM-induced platelet hyperreactivity is presented in Figure 2.

## 4. Platelet Glycoprotein Receptor-ib (GPIb)

During the pathological process, platelets play an important role in vascular interactions through their receptors [51,52]. Platelet membrane glycoproteins have an important role as receptors in two key processes: attachment to the subendothelial matrix and platelet aggregation [34,53,54]. As mentioned earlier, the atherosclerosis process is initiated by the adhesion step; whereas platelets adhere to intact endothelial cells, the process of transitioning from a passive circulating state to an active adhesive state in the extracellular matrix is regulated by platelet membrane receptors [9,25]. GPIb/IV/V, GPVI, and GPIa/IIa, along with other vital ligands, facilitate this adhesion [51,52]. When ligand-receptor engagement occurs, signaling mechanisms are activated, leading to changes in calcium oscillation, agonist molecule release, and platelet degranulation, which in turn induce events that lead to the activation of other platelets and the formation of a stable clot [51,52]. Among other platelet glycoprotein adhesion receptors, the GPIb/IX/V receptor complex represents the second-most expressed adhesion receptor found on platelets [22,23,24]. It comprises around 25,000 units of the GPIb-IX complex and 12,000 GPV units on the resting platelets [55].

**Figure 3:**
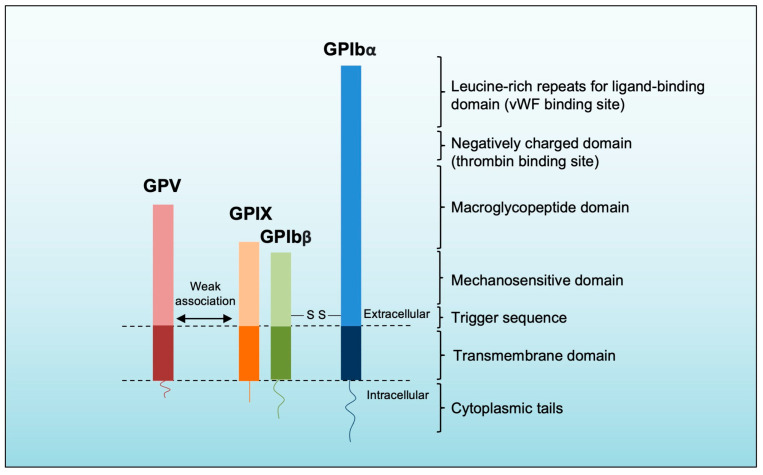
Platelet GPIb/IX/V’s structure. The largest subunit of the complex is GPIbα, comprising leucine-rich repeats in the ligand-binding domain, a negatively charged domain, a macroglycopeptide domain, a mechanosensitive domain, and the trigger sequence. GPIbα and GPIbβ are connected through disulfide bonds. The transmembrane domain facilitates close interaction with GPIbβ and GPIX, forming a stable parallel structure. GPV weakly interacts with GPIb-IX through polar interactions and can potentially be replaced by other receptors.

Platelet GPIb/IX/V’s structure has been well-reviewed elsewhere [24,56]. Briefly, GPIb/IX/V is a protein complex comprising three components: GPIbα-GPIbβ, GPIX, and GPV (Figure 3) [24]. The GPIbα, GPIbβ, and GPIX cluster into the GPIb/IX complex on the platelet membrane [24,57]. GPIbα is the largest one and mediates interactions with many known ligands [24]. It contains eight LRR that form the ligand-binding domain (LBD) and a negatively charged portion with three tyrosine residues with sulfate groups crucial for thrombin binding [24,56,58]. Additionally, GPIbα has a mucin-like macroglycopeptide region that aids in ligand-receptor complex formation [56]. The mechanosensitive domain (MSD), another identified domain in GPIbα, is located next to the macroglycopeptide region and can be cleaved by ADAM17, releasing soluble GPIbα fragments into the plasma [24,59]. Recently, a trigger sequence of 10 amino acid residues has been discovered near the transmembrane domain of GPIbα [56]. The discovery provides support for the GPIb-IX signaling model, proposing that when external forces act on the LBD of GPIbα, it induces the unfolding of the mechanosensitive domain (MSD) [56]. This unfolding exposes a trigger sequence, resulting in receptor activation and the subsequent initiation of signaling pathways within the platelet [56]. The other components of GPIbα-GPIbβ, the GPIbβ, make disulfide bonds with GPIbα and are connected to GPIX [56]. GPIbβ engages in intracellular interaction with calmodulin, whereas GPIX does not form associations with any intracellular molecules [24,57]. Moreover, the cytoplasmic tail of GPIbα possesses multiple binding sites for intracellular signaling molecules, facilitating the connection of the receptor complex to actin filaments within the cytoskeleton [24]. Interactions between the transmembrane domains of the components, including the engagement of GPIbβ and GPIX with the mechanosensitive domain (MSD) of GPIbα, stabilize the GPIb-IX complex [24]. The association of GPV with the complex is relatively weak, and its susceptibility to non-ionic detergents suggests that the GPIb/IX complex can potentially interact with other membrane receptors along with GPV [24].

## 5. Platelet GPIb Hyperreactivity in T2DM and Its Role in the Pathogenesis of Atherosclerosis

The activation, adhesion, and aggregation of platelets are crucial processes significantly contributing to advanced atherosclerosis-induced cardiovascular disease, leading to serious clinical events of CAD such as myocardial infarction or heart attack [3,9,10]. These events occur due to the aggravation of atherosclerosis, which happens when endothelial plaque is disrupted, causing platelet adhesion, activation, and thrombus formation [3,9,10]. Patients with T2DM are at great risk of having cardiovascular diseases due to platelet hyperreactivity, which is common in T2DM patients with hyperglycemia [3,27,28]. Atherosclerosis is a condition where the walls of the arteries narrow due to lipid and cholesterol accumulation [41,60]. In patients with T2DM, changes in the coronary artery wall, platelet hyperreactivity, and fibrin deposition can cause progressive narrowing of the artery lumen [41,60]. These changes may lead to a sudden disruption of blood flow due to plaque rupture with thrombosis [41,60]. This can exacerbate the narrowing of the artery lumen, compromising blood flow and potentially leading to serious cardiovascular incidents [41,60].

T2DM has been marked as a chronic disease with prothrombotic status because it has shown platelet changes and coagulation characteristics [37,61,62]. Individuals with T2DM often have increased platelet adhesiveness, which is associated with higher levels of adhesive molecules such as the platelet receptors GPIb/CD42 and vWF [29,63,64,65,66,67,68]. The significance of platelets in vascular occlusion and thromboinflammation is strongly linked with their adherence capacity to injured endothelial cells and immune cells [34,69,70]. T2DM eventually precipitates endothelial dysfunction and magnifies inflammation, vasoconstriction, and thrombosis [62]. A study shows that in mice with streptozosocin-induced diabetes, platelets have more frequent interactions with endothelial vessels. This interaction is due to increased expression of vWF in endothelial cells and is mediated by platelet GPIb-IX-V [71].

The platelet GPIb/IX receptor complex is essential in regulating normal and pathological platelet processes [25]. For example, during the hemostasis process, the sequestration of platelets at the site of vascular injury is initiated through the interaction between platelet GPIb/IX and the vWF factor on the subendothelial matrix [25,72]. When there is damage to the endothelial layer of blood vessels, subendothelial collagen and other components are exposed, leading to platelet adhesion and activation [25,72]. The initial interaction is between the platelet GPIbα and vWF, and their interaction initiates platelet adhesion and triggers a signaling cascade that induces platelet integrin αIIbβ3 activation [25,72]. Activated αIIbβ3 then allows fibrinogen binding, leading to platelet aggregate formation [25]. Moreover, vWF can also be activated by high shear forces and becomes more adherent to the platelet GPIbα receptor, which further activates platelets and enhances thrombus formation [25,72].

In addition, the link between GP1b and vWF induces thromboxane A2 production, resulting in ADP secretion and fibrinogen receptor activation, the key steps in platelet activation [25,72]. The GP1b-IX-mediated platelet activation occurs through various signaling pathways, including the mitogen-activated protein kinase pathways, the phosphatidylinositol 3-kinase (PI3-kinase) protein kinase B (Akt) pathway, the FcRγ-Syk/PLCγ2 pathway, and the LIM kinase 1 (LIMK1) pathway [73,74]. After establishing adhesion, the platelets become more activated and generate more pro-inflammatory molecules and chemoattractants [31]. Platelets’ adhesion to the vascular endothelial cells delivers messages for leukocyte engagement and monocyte extravasation [31]. In atherosclerosis, the protective role of the vascular endothelium diminishes, resulting in an increased number of activated platelets [31]. Subsequently, the activated platelets produce more inflammatory molecules, generating chronic inflammation in the endothelium and resulting in vascular and cellular dysfunction [31].

The interaction between the platelet receptor GPIbα and the leukocyte integrin macrophage-1 antigen (Mac-1) also plays a role in thrombosis [75]. This interaction facilitates the mobilization, adherence, and migration of leukocytes to vascular lesion sites, promoting vascular inflammation [75]. Moreover, thrombus formation is entirely abolished in studies that use GPIb knockout mice, highlighting the crucial role of the GPIb receptor’s interaction with vWF in platelet adhesion and platelet interaction with immune cells. [25,75,76]. Additionally, apart from vWF, other ligands for the GPIb receptor might play a pivotal role in platelet aggregation and thrombosis, significantly contributing to platelet adhesion [25]. The GPV receptor interacts with additional ligands and counter-receptors within the bloodstream, participating in diverse aspects of platelet biology that are still under investigation [25]. Platelet GPIb receptors are important molecules during atherosclerosis’s early and later stages. Research indicates that inhibiting the interaction between GPIbα and vWF can have a protective effect by reducing the occurrence of microvascular blockages and improving the patency of blood vessels. [26]. As a result, several agents are currently being investigated to target the platelet adhesion signaling pathway by inhibiting GpIb for potential therapeutic applications.

## 6. Targeting GPIb as a Potential Therapy May Protect T2DM Patients from Developing Atherosclerosis-Induced Cardiovascular Diseases

Since patients with T2DM have demonstrated higher atherogenesis and atherothrombotic complications, antithrombotic drugs are needed as management therapy for these individuals [10,17,18,44,77]. Current treatment strategies for individuals with high risk of CAD, such as T2DM patients, primarily target enhancing vascular outcomes and revolve around restoring blood flow in obstructed arteries [10,17,18,78]. Therefore, antiplatelet agents are needed to prevent platelets from clumping and clotting from forming and growing in patients with T2DM [10,17,18,78]. Over the years, two primary targets for antithrombotic therapy have been extensively employed. These include using cyclooxygenase-1 (COX-1) inhibitors, aiming to diminish the production of TXA2 (e.g., low-dose aspirin), and utilizing platelet P2Y12 receptor antagonists (e.g., clopidogrel, prasugrel, ticagrelor, cangrelor, and elinogrel) [10,17,18,78]. However, despite their widespread use, the rates of recurrent atherothrombotic events remain high, particularly in diabetic patients, indicating inadequate protection against cardiovascular events [16,17,18,19,20]. The concept of antiplatelet drug resistance is relevant to aspirin and clopidogrel [16,17]. Furthermore, a randomized trial has suggested that aspirin may not effectively reduce the risk of cardiovascular events in primary prevention and could potentially increase the risk of gastrointestinal bleeding [16,79]. The variability in platelet response to clopidogrel can be attributed to genetic, cellular, and clinical factors [16,17,80,81]. Notably, diabetic patients, especially those requiring insulin therapy, are more likely to be nonresponsive to clopidogrel [16,17]. Additionally, dual antiplatelet therapy (DAPT), which combines aspirin with P2Y12 inhibitors (primarily clopidogrel), is commonly used in patients with CAD and those undergoing percutaneous coronary intervention (PCI), regardless of their T2DM status [82,83]. However, it is worth noting that reports of reduced responsiveness to DAPT and an increased risk of bleeding have been described [17,84,85]. Another class of antiplatelet agents used to reduce cardiovascular complications associated with PCI is GPIIb/IIIa antagonists, which inhibit platelet aggregation [10,17,25]. Currently, three intravenous GPIIb/IIIa antagonists are available: the monoclonal antibody abciximab, as well as the small molecules eptifibatide and tirofiban [10,17,25]. It is important to consider that the use of GP IIb/IIIa antagonists has been associated with an increased risk of thrombocytopenia and bleeding complications [4,17,20,86].

Collectively, it underscores the need for newer targeted antiplatelet treatment approaches in diabetic patients. This may involve utilizing more potent medications or combining different antiplatelet drugs to enhance effectiveness [4,16,17,18]. In the future, antiplatelet drug regimens could be utilized based on a “stage-specific” vascular management strategy and personalized needs [4,16,17,18]. The most promising strategy to accomplish this objective is to develop new antiplatelet agents that specifically target pathways commonly observed in a specific patient population, such as elevated GPIb levels in patients with T2DM [4,25,87,88].

Researchers have conducted several studies to develop drugs targeting the GPIb receptor, subsequently decreasing platelet adhesion and preventing thrombosis [10,25,87,89,90]. One study by David et al. utilized an inhibitory peptide called R9α557 that can penetrate the cell membrane and contains nine arginine amino acid residues to facilitate entry into the cell [25,91]. The peptide comprises a sequence of 11–13 amino acids from the cytoplasmic region of the GPIb receptor [25,91]. Further, the study demonstrated that the R9α557 peptide could effectively reduce vWF-mediated adhesion in human platelets [25,91]. Another experimental approach involved using a monoclonal antibody, 6B4, that targeted the GPIb receptor in nonhuman primates, as conducted by Cauwenberghs et al. [25,92]. The findings from this study showed that the monoclonal antibody could reduce thrombus formation without causing significant prolongation of bleeding time [25,92]. Additionally, Zahger et al. conducted a study in which they utilized VCL, a recombinant von Willebrand factor GP1b binding domain, as an antagonist to the GPIb receptor in rats [25,93]. Their investigation provided compelling evidence that using VCL, a platelet GP1b receptor antagonist, yielded notable reductions in platelet adhesion and the extent of intimal thickening following balloon injury to the femoral artery in rats [25,93].

Another interesting study involved compounds derived from snake venom called C-type lectins, which have generated mixed results in various experimental settings [25,87,94,95]. While some agents have led to platelet inhibition, others have resulted in platelet activation [25,87,94,95]. However, a group of researchers focused on one particular compound called anfibatide, a snake venom-derived GPIb antagonist [93,96,97,98]. They successfully synthesized it using recombinant technology and tested its ability to inhibit the GPIbα-vWF interaction in vitro and in vivo studies [96,99]. Their finding also indicates that the researchers developed a capable method of generating recombinant anfibatide in substantial quantities [96,99]. This approach aimed to overcome the challenges of purifying anfibatide from raw snake venom and the limited supply of this natural resource [96,99]. Next, after accumulating positive in vitro and in vivo results, the researchers evaluated the safety and efficacy of anfibatide in healthy human individuals [96,100,101,102,103]. Their investigations yielded compelling evidence demonstrating the specific inhibition of GPIbα-vWF interaction and associated platelet functions by anfibatide [96,103]. Notably, this inhibition was observed in various scenarios, such as ferric chloride- and laser-induced thrombus formation in mesenteric and cremaster muscle arterioles [96,103]. The positive outcomes prompted subsequent studies involving human subjects [96,103]. These results strongly suggest that anfibatide possesses selective antithrombotic properties targeting GPIb in humans [96,103]. This noteworthy finding was briefly mentioned during the American Society of Hematology’s 2013 annual meeting [96,103]. In managing and treating CAD characterized by elevated shear stress, anfibatide may emerge as a more favorable alternative to αIIbβ3 and vWF antagonists [96,103]. This results from the pivotal role of the GPIbα-vWF interaction in thrombosis under such conditions [96,103]. Consequently, utilizing anfibatide could potentially improve patients’ risk/benefit ratio in these scenarios [96,103]. Significantly, this marked the first instance of testing a GPIbα antagonist in humans, as registered in ClinicalTrials.gov. The outcomes from the Phase I study indicate that anfibatide may serve as a safe and effective therapeutic agent for antithrombotic therapy, specifically targeting platelet GPIbα [96,103].

Moreover, the study revealed a promising safety profile for anfibatide, providing a foundation for advancing to subsequent clinical trial phases: Phases Ib-IIa and Phase II [96]. Anfibatide holds potential as a therapeutic agent for treating CAD, with particular relevance for patients with T2DM who face an increased risk of cardiovascular complications accompanied by elevated levels of GPIb [93,96,100,101,102,103]. Further investigations are warranted to determine optimal dosing strategies and evaluate the safety profile for patients with a high intracoronary thrombus burden. Nonetheless, anfibatide exhibits promise as an anti-GPIb agent, offering prospects for enhanced treatment options.

## 7. Conclusions

**Figure 4:**
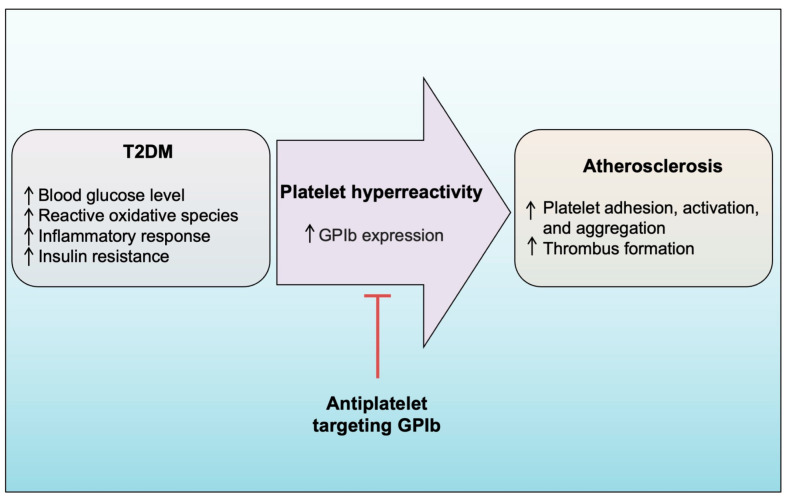
Platelet glycoprotein-Ib (GPIb) may act as a bridge between T2DM and atherosclerosis. In the context of T2DM, various risk factors such as high glucose levels, insulin resistance, oxidative stress, and inflammation can contribute to increased platelet hyperreactivity. One of the effects of these risk factors is the upregulation of GPIb expression, which promotes platelet adhesion, activation, and aggregation. These processes, in turn, contribute to the development and progression of atherosclerosis.

Platelet adhesion and aggregation are important in preventing excessive bleeding during tissue injury, including vascular injury. These processes are particularly significant in vascular thrombosis, such as in cerebral arteries or atherosclerotic coronary vessels, which can result in stroke, heart attack, and lower extremity PAD. Numerous studies have reported that individuals with T2DM face a high risk of atherosclerotic cardiovascular disease. To manage cardiovascular thrombotic disorders in patients with T2DM, antiplatelet agents such as aspirin, GPIIb/IIIa inhibitors, and clopidogrel are currently used as treatment options. However, recent studies have highlighted the limitations of these therapies, including resistance issues and severe side effects, indicating the need for newer antiplatelet agents. Studies have shown that individuals with T2DM often experience platelet hyperreactivity and a prothrombotic state linked to increased expression of various platelet glycoproteins, including GPIb. When exposed to strong shear forces, GPIb facilitates platelet adhesion by interacting with vWF. Subsequently, the adhered platelets become activated and aggregate, forming a hemostatic plug or an occlusive thrombus. Collectively, these findings highlight the crucial role of GPIb as a bridge connecting T2DM and atherosclerosis (Figure 4). Therefore, this review article emphasizes the discovery of the first antiplatelet agents targeting platelet GPIb, which have progressed to clinical trial studies, such as anfibatide. Anfibatide has demonstrated promising antiplatelet effects and a low bleeding tendency in clinical trials. Given that GPIb is an attractive target for attenuating thrombosis and its expression is heightened in patients with T2DM, anfibatide could be an effective antiplatelet agent to prevent cardiovascular disease complications in this specific population. Nevertheless, further studies and trials, encompassing experimental in vitro and in vivo investigations, including clinical trials, are necessary to thoroughly evaluate the effectiveness of anfibatide in achieving a higher antiplatelet effect, particularly in T2DM-specific populations.

## Data Availability

Not applicable.
